# A study of motivations and expectations of patients seen in phase 1 oncology clinics

**DOI:** 10.1002/cncr.30235

**Published:** 2016-09-26

**Authors:** Saoirse O. Dolly, Eleftheria Kalaitzaki, Martina Puglisi, Sarah Stimpson, Janet Hanwell, Sonia Serrano Fandos, Sarah Stapleton, Thushara Ansari, Clare Peckitt, Stan Kaye, Juanita Lopez, Timothy A. Yap, Winette van der Graaf, Johann de Bono, Udai Banerji

**Affiliations:** ^1^ Department of MedicineRoyal Marsden NHS Foundation TrustLondonUnited Kingdom; ^2^ Division of Clinical StudiesInstitute of Cancer ResearchLondonUnited Kingdom; ^3^ Clinical R&DRoyal Marsden NHS Foundation TrustLondonUnited Kingdom

**Keywords:** cancer, oncology clinical trials, patient expectations, patient motivations, phase 1 trials

## Abstract

**BACKGROUND:**

To better inform clinical practice, this study was aimed at capturing patients' motivations for enrolling in phase 1 trials and at quantifying their expectations of the benefits, risks, and commitment associated with clinical trials and the impact of the initial consultation on their expectations.

**METHODS:**

This was a single‐center, prospective, quantitative study of newly referred adult patients considering their first phase 1 oncology trial. Participants completed questionnaires before they were seen and an abbreviated follow‐up version after their consultation.

**RESULTS:**

Questionnaires were completed by 396 (99%) and 301 (76%) before and after the clinic, respectively. Participants ranked the possibility of tumor shrinkage (84%) as the most important motivation for considering a phase 1 trial; this was followed by no alternative treatments (56%), their physician's recommendation (44%), and the fact that the research might benefit others (38%). When they were asked about the potential personal benefit, 43% predicted tumor shrinkage initially. After the consultation, this increased to 47%. Fourteen percent of patients expected a cure. When asked about risks, 71% of the participants expected moderate side effects. When asked about expectations of time commitments, a majority of patients did not anticipate weekly visits, although this was understood by 93% of patients after the consultation. Overall, patients were keen to consider trials and when asked before and after the consultation 72% and 84% were willing to enroll in studies, respectively.

**CONCLUSIONS:**

This study reports that more than 80% of patients enroll in early‐phase clinical oncology trials motivated by the potential of a clinical benefit, with approximately half expecting tumor shrinkage and approximately a tenth anticipating a cure. The typical phase 1 response rate is 4% to 20%, and this discrepancy exemplifies the challenges faced by patients and healthcare professionals during their interactions for phase 1 studies. ***Cancer* 2016;122:3501–3508**. © *2016 American Cancer Society*

## Abstract 2

More than 80% of patients seen in phase 1 oncology clinics are willing to participate in studies, with approximately half hoping for tumor shrinkage, and approximately a tenth anticipating a cure. These exceed realistic response rates of 4% to 20%, and the initial phase 1 consultation does not alter the majority of expectations except for an understanding of the time commitment involved.

## INTRODUCTION

Phase 1 trials are designed to recommend the dose and schedule for a novel anticancer drug, with the toxicity as well as the pharmacokinetic and pharmacodynamic profile taken into account. These first‐in‐human studies often start at conservatively low doses of the new drug and escalate the dose until a recommended phase 2 dose is established. Thus, patients in phase 1 trials can receive subtherapeutic doses with little realistic chance of efficacy or an excessively high drug dose with a risk of serious toxicity. Several studies over the past 20 years have quantified typical response rates of phase 1 trials as 4% to 20% with a median overall survival of 6 months.^1^, ^2^, ^3^

First‐in‐human oncology drug trials are normally restricted to patients with advanced malignant disease refractory to standard therapy because of the narrow therapeutic indices of anticancer drugs. This is a unique group of patients who have usually received several lines of previous treatments, have a short life expectancy,^4^ and have few remaining conventional treatment options. Entry into a phase 1 study requires the judgment of the relative likelihood of benefit versus toxicity and is essentially an individual patient's decision.^5^, ^6^ This encompasses a patient's motivations, expectations, and ability to comprehend the complexities of clinical trial research.^7^, ^8^ Ethically, healthcare professionals must ensure that patients are well informed about the likelihood of risks and benefits, have a good understanding of the trial, and know the alternatives to study participation to aid the decision‐making process.^9^, ^10^, ^11^

Research into patients' perceptions has generally focused on participants who have already enrolled or who are considering entry into early‐phase clinical trials, that is, after discussions have already taken place.^8^, ^12^, ^13^, ^14^, ^15^ Patients have demonstrated unrealistically high expectations of personal benefit or even a cure from these investigational agents.^15^, ^16^, ^17^ Much of the research has attributed these idealistic expectations to suboptimal patient‐physician communication and a patient‐based culture of needing to remain optimistic as treatment options diminish.^11^, ^12^, ^14^, ^15^, ^18^

It is important to quantify how effective communication is with regard to these phase 1 discussions.^11^ This is an iterative process over multiple appointments at which trial‐specific information sheets and consent forms are discussed with patients and their families. However, communication regarding the general concepts of phase 1 studies during the first outpatient visit is critical and influences whether patients proceed with the trial process. Currently, there is a paucity of quantitative research assessing how patients' preconceptions change before and after the clinical consultation.^15^, ^19^ Pre‐ and postconsultation questionnaires using a quantitative assessment of patient's expectations and perceptions would facilitate improved communication by the clinical team. If specific groups were found to have significantly different perceptions, such information could be used to tailor discussions to these groups.

To the best of our knowledge, no studies have previously assessed patients' motivations and perceptions before they are seen by the clinical trial team in the setting of phase 1 oncology studies. The aim of this study was to explore patients' motivations for considering phase 1 trials and to assess the different factors influencing their participation. This study also aimed to quantify patients' expectations before they were reviewed in clinic and whether these changed immediately after the consultation. To judge patient optimism, expectations on cure rates were assessed only with the postconsultation questionnaire because this was felt to be too sensitive to be posed before they met their oncologist.

## MATERIALS AND METHODS

### Procedures and Measures

The Committee for Clinical Research of the Royal Marsden Hospital and the Institute of Cancer Research (CCR no. 3745), along with the research ethics committee, approved the study and questionnaires.

This was a prospective, non‐randomized, quantitative study undertaken at the Phase 1 Drug Development Unit of the Royal Marsden Hospital in Sutton, United Kingdom. The study aims were 4‐fold: 1) to capture patients' motivations for phase 1 clinical trials and assess whether they were influenced by age, sex, education, or cancer type; 2) to quantify baseline expectations of the benefits, risks, and commitments associated with clinical trials; 3) to assess how the consultation affected patients' perceptions; and 4) to quantify the expectations of a cancer cure from these experimental trials.

Patient information sheets were sent with the appointment letter. After registration, patients wishing to participate signed the consent form. They completed a preclinic questionnaire before they were reviewed by the clinical team (Supporting Information 1 [see online supporting information]). After the consultation, they completed their postclinic questionnaire (Supporting Information 2 [see online supporting information]) in private.

### Participants

Prospective patients who were attending the phase 1 trial outpatient clinic for the first time were recruited. Eligible patients were 18 years or older and were able to provide informed written consent in English. The only exclusion criterion was prior phase 1 trial participation. An *a priori* sample size calculation was used. All new patients in the clinic were invited to participate to prevent any selection bias.

### Questionnaires

A multidisciplinary team including medical oncologists, nurses, clinical trial coordinators, and statisticians devised the study questionnaires. These were refined after a small pilot study and input from the Royal Marsden Patient Advocacy Group (Supporting Information 1 and 2 [see online supporting information]).

The preconsultation questionnaire (Supporting Information 1 [see online supporting information]) comprised 2 sections. Section 1 captured the baseline demographics, referral route, and patient motivations for phase 1 trials. Participants independently ranked motivation on a scale of 1 to 5 of how important 5 factors were to their participation in the trial: 1) “shrink tumor,” 2) “no other treatment options available,” 3) “family wishes,” 4) “research will benefit others,” and 5) their referring “doctor recommended it.” Section 2 focused on expectations of the benefits, risks, and commitment associated with phase 1 trials. The benefits included expectations of a tumor response (shrinkage, no change, growth, or do not know), and the risks included perceived side effects (mild, moderate, or severe) and, if applicable, a comparison with chemotherapy (better, same, or worse). Other factors included the commitment: the believed frequency of hospital visits (weekly, monthly, or less than monthly) and the willingness to participate in a phase 1 trial (yes, no, or do not know).

The postconsultation questionnaire (Supporting Information 2 [see online supporting information]) repeated section 2. In addition, a further sensitive question was posed: “Do you expect your cancer will be cured on a phase 1 trial?” All responses were anonymized, and data were double‐checked on entry into a database.

### Statistical Considerations

All available data were used, and descriptive statistics were used to summarize patient demographics, clinical characteristics, and question responses. Cross‐tabulations of the before and after questionnaires used the Stuart‐Maxwell test statistic for marginal homogeneity. Univariate and multivariate logistic regression analyses were undertaken to explore whether age, sex, education level, and tumor type represented independent variables influencing patients' perceptions of phase 1 clinical trials with respect to benefits, risks, time commitment, and willingness to participate. All patients with complete sets of data were included in the multivariate models, which were adjusted for the effects of all the variables without any prior selection. Analyses were conducted with Stata 13.1.

## RESULTS

### Patient Characteristics

Between September 2012 and March 2015, questionnaires were given to 402 prospective adult patients at their first attendance in the new patient phase 1 clinic of the Drug Development Unit. Ninety nine percent (n = 396) gave written consent to participate and completed the preclinic questionnaire; 301 patients returned the postclinic questionnaire (76%). The mean age was 57.4 years (standard deviation, 12.9 years); there was a slight preponderance of females (56.3%), and there was an even distribution of participants leaving formal education before and after the age of 17 years (46.2 vs 44.7%; Table 1). The commonest cancers were gastrointestinal, gynecological, and lung cancers, with their respective cancer specialists being the major primary referrers for 87.1% of the patients (Table 1).

**Table 1:** Characteristics of the Study Participants

Characteristic	Value
Age, mean ± standard deviation (range), y	57.4 ± 12.9 (19.3‐67.4)
Sex, No. (%)	
Male	173 (43.7)
Female	223 (56.3)
Total	396 (100.0)
Cancer site, No. (%)	
Breast	40 (10.1)
Gastrointestinal	153 (38.6)
Gynecological	66 (16.7)
Lung	58 (14.6)
All others	79 (19.9)
Total	396 (100.0)
Level of education, No. (%)	
Left education at < 17 y	183 (46.2)
Left education at ≥ 17 y	177 (44.7)
Missing	36 (9.1)
Total	396 (100.0)
Referrer, No. (%)	
Cancer specialist	345 (87.1)
General practitioner	4 (1.0)
Family/friends	7 (1.8)
Internet	12 (3.0)
Other	16 (4.0)
Unknown	12 (3.0)
Total	396 (100.0)

#### Baseline patient motivations for early clinical trials

Factors that patients considered the most and least important for entering a phase 1 trial are summarized in Table 2. Each question was answered independently; this meant that they could rank more than 1 question with the same score. The most important motivation was shrinkage of their tumor (83.5%), which was followed by no alternative treatments (56.0%), their referring physician's recommendation (44.2%), the benefit of research to others (38.0%), and family wishes (24.4%).

**Table 2:** Patients' Independently Ranked Most Important Baseline Reasons for Considering Phase 1 Trials

Baseline Reason	Responses, No. (%)				
	1 (Most Important)	2	3	4	5 (Least important)
Tumor shrinkage (n = 358)	299 (83.5)	29 (8.1)	14 (3.9)	5 (1.4)	11 (3.1)
No other treatment options (n = 332)	186 (56.0)	65 (19.6)	46 (13.9)	15 (4.5)	20 (6.0)
Family wishes (n = 312)	76 (24.4)	40 (12.8)	89 (28.5)	46 (14.7)	61 (19.6)
Benefit of research to others (n = 337)	128 (38.0)	85 (25.2)	70 (20.8)	35 (10.4)	19 (5.6)
Physician's recommendation (n = 330)	146 (44.2)	65 (19.7)	65 (19.7)	24 (7.3)	30 (9.1)

Univariate analyses and multivariate analyses (MVAs) were undertaken to determine the most important reasons for entering a phase 1 trial according to age, education, sex, and tumor type (Table 3 and Supporting Information 3 [see online supporting information]). Gastrointestinal cancers, the largest group, were used as the reference category in the cross‐tumor comparisons. The most statistically significant factors that affected patient participation were related to age and education. Interestingly, older patients (≥59.5 years) were more dependent on their referring physician's recommendation (odds ratio [OR] from MVA model, 1.98; 95% CI [confidence interval], 1.19‐3.30; *P* = .01) and the benefit of research (OR from MVA model, 1.91; 95% CI, 1.15‐3.18; *P* = .01). Participants who left education before the age of 17 years were more likely to deem tumor shrinkage (OR from MVA model, 0.37; 95% CI, 0.15‐0.94; *P* = .04), a lack of alternative treatments (OR from MVA model, 0.56; 95% CI, 0.32‐0.98; *P* = .04), family wishes (OR from MVA model, 0.46; 95% CI, 0.27‐0.76; *P* < .001), and the benefit of research (OR from MVA model, 0.51; 95% CI, 0.31‐0.83; *P* = .01) as the most important reasons for considering an early‐phase clinical trial. With the exception of “no other treatments available,” the cancer type was not significant in the remaining analyses. The response “no other treatments available” was highly significant (*P* = .001), and this meant patients with gastrointestinal cancers were more likely to answer that they were interested in phase 1 trials because there were no alternatives. Within this subgroup (n = 135), 83% of the patients replied that this was the most important reason; the OR within this group was 4.87 (95% CI, 3.11‐7.63; *P* < .001). There was a similar effect within the “other” cancer group (n = 71) with an OR of 5.45 (95% CI, 2.87‐10.37; *P* < .001).

**Table 3:** Univariate and Multivariate Logistic Regression Analyses Assessing Participants' Motivations for Phase 1 Oncology Trials According to Age, Education, Sex, and Tumor Type

Patient Motivation to participate in Phase 1 Trials	Sociodemographic Variables	Univariate Analysis				Multivariate Analysis			
		OR	*P*	95% CI		OR	*P*	95% CI	
Tumor shrinkage	Age (≥59.5 vs < 59.5 y)	1.75	.16	0.81	3.79	1.16	.74	0.49	2.74
	Education (≥17 vs < 17 y)	0.34	**.02**	0.14	0.83	0.37	**.04**	0.15	0.94
	Sex (female vs male)	1.28	.52	0.60	2.70	0.81	.66	0.32	2.08
	Breast vs GI	1.38	.69	0.29	6.61	1.59	.59	0.30	8.59
	Gynecological vs GI	1.16	.81	0.35	3.85	1.80	.42	0.43	7.59
	Lung vs GI	2.03	.37	0.43	9.61	1.74	.49	0.36	8.52
	Other vs GI	0.43	.07	0.18	1.06	0.52	.20	0.20	1.40
No other treatment options available	Age (≥59.5 vs < 59.5 y)	1.59	.07	0.96	2.65	1.50	.17	0.84	2.68
	Education (≥17 vs < 17 y)	0.55	**.02**	0.32	0.92	0.56	**.04**	0.32	0.98
	Sex (female vs male)	0.90	.69	0.54	1.49	2.20	**.03**	1.08	4.46
	Breast vs GI	0.31	**.01**	0.13	0.73	0.25	**.01**	0.09	0.69
	Gynecological vs GI	0.35	**.00**	0.17	0.71	0.23	**.00**	0.10	0.57
	Lung vs GI	0.37	**.01**	0.17	0.79	0.32	**.01**	0.14	0.73
	Other vs GI	1.12	.78	0.51	2.45	1.34	.48	0.59	3.03
Family wishes	Age (≥59.5 vs < 59.5 y)	2.17	**.00**	1.36	3.47	1.62	.07	0.96	2.75
	Education (≥17 vs < 17 y)	0.42	**.00**	0.26	0.68	0.46	**.00**	0.27	0.76
	Sex (female vs male)	1.05	.83	0.66	1.67	1.36	.30	0.76	2.43
	Breast vs GI	0.52	.15	0.22	1.26	0.51	.20	0.18	1.42
	Gynecological vs GI	0.62	.18	0.31	1.25	0.54	.13	0.24	1.20
	Lung vs GI	1.71	.13	0.85	3.44	1.23	.60	0.57	2.66
	Other vs GI	0.92	.78	0.49	1.71	1.04	.91	0.53	2.04
Benefit of research to others	Age (≥59.5 vs < 59.5 y)	2.14	**.00**	1.36	3.37	1.91	**.01**	1.15	3.18
	Education (≥17 vs < 17 y)	0.45	**.00**	0.28	0.72	0.51	**.01**	0.31	0.83
	Sex (female vs male)	1.04	.85	0.67	1.63	1.15	.64	0.64	2.05
	Breast vs GI	0.81	.60	0.38	1.75	1.06	.90	0.43	2.59
	Gynecological vs GI	0.77	.42	0.41	1.45	0.82	.61	0.39	1.75
	Lung vs GI	1.42	.35	0.68	2.96	1.14	.74	0.52	2.49
	Other vs GI	0.79	.45	0.43	1.45	1.03	.93	0.53	1.99
Referring physician's recommendation	Age (≥59.5 vs < 59.5 y)	2.24	**.00**	1.41	3.56	1.98	**.01**	1.19	3.30
	Education (≥17 vs < 17 y)	0.67	.09	0.42	1.06	0.75	.26	0.46	1.23
	Sex (female vs male)	1.12	.62	0.71	1.77	1.44	.22	0.81	2.58
	Breast vs GI	0.76	.48	0.35	1.63	0.80	.63	0.33	1.94
	Gynecological vs GI	0.98	.94	0.51	1.86	0.83	.63	0.39	1.76
	Lung vs GI	1.79	.13	0.85	3.79	1.56	.28	0.70	3.49
	Other vs GI	1.07	.83	0.58	1.99	1.13	.73	0.58	2.19

Abbreviations: CI, confidence interval; GI, gastrointestinal; OR, odds ratio.

ORs greater than 1 indicate that older patients (≥59.5 years), more educated patients (left formal education at an age ≥ 17 years), females (vs males), and patients with breast, gynecological, lung, or other tumors (vs gastrointestinal cancers) were more likely to rate high the listed reasons for considering an early‐phase clinical trial. NB, *P* values in bold are statistically significant (*P* < 0.05).

#### Patients' baseline expectations of the benefits, risks, and commitment of phase 1 trials

The next analysis assessed what patients' expectations were of a phase 1 study before they were seen in the clinic (Table 4).

**Table 4:** Patients' Perceptions of the Benefits, Risks, Commitment, and Cure Associated With Phase 1 Trials on the Pre‐ and Postconsultation Questionnaires

Question	Response	Preconsultation Questionnaire (n = 396)	Postconsultation Questionnaire (n = 301)
Benefit: tumor response, No. (%)	Available data	384 (97.0)	301 (100)
	Shrink	164 (42.7)	141 (46.8)
	Same	24 (6.3)	32 (10.6)
	Grow	1 (0.3)	4 (1.3)
	Do not know	195 (50.8)	124 (41.2)
Risks, No. (%)			
Side effects	Available data	351 (88.6)	288 (95.7)
	Mild	63 (17.9)	46 (16.0)
	Moderate	248 (70.7)	222 (77.1)
	Severe	40 (11.4)	20 (6.9)
Side effects vs chemotherapy	Available data	366 (92.4)	296 (98.3)
	Better	91 (24.9)	97 (32.8)
	Same	223 (60.9)	160 (54.1)
	Worse	38 (10.4)	29 (9.8)
	No prior chemotherapy	14 (3.8)	10 (3.4)
Commitment, No. (%)			
Hospital visits	Available data	340 (85.9)	299 (99.3)
	Once a week	169 (49.7)	278 (93.0)
	Once a month	153 (45.0)	19 (6.4)
	Less than monthly	18 (5.3)	2 (0.7)
Willingness to enter trial	Available data	383 (96.7)	299 (99.3)
	Yes	274 (71.5)	252 (84.3)
	No	2 (0.5)	4 (1.3)
	Do not know	107 (27.9)	43 (14.4)
Cure: expectation that trial will cure cancer, No. (%)	Available data	Not applicable	300 (99.7)
	Yes	Not applicable	42 (14.0)
	No	Not applicable	146 (48.7)
	Do not know	Not applicable	112 (37.3)

##### Benefits

 Among those patients with data (n = 384), approximately 42.7% thought that their tumors would shrink, whereas 51% stated that they did not know.

##### Risks

 When the patients were asked to rate what kinds of side effects there would be, the most common answer was moderate (70.7%). When they were asked how these would compare with chemotherapy, the commonest answer was the same (60.9%) for those who had received prior chemotherapy (n = 286).

##### Commitment

Approximately half of the patients (49.7%) thought they would be seen weekly, with 45.0% expecting once monthly visits. Notably, 71.5% of the patients expected to enter a phase 1 study at this stage, and 27.9% marked “do not know” and were thus undecided at this point.

#### Changes in patients' perceptions after the clinic consultation

The final analysis explored how patients' understanding of phase 1 trials changed after the detailed new patient clinic discussion (Table 4).

##### Benefit

The expectation of tumor shrinkage increased in the postclinic questionnaire to 46.8% from 42.7%.

##### Risks

The most frequent toxicity response was still “moderate” (77.1% from 70.7%); however, this was not significant (*P* = .16). There was a small but significant change in the perception of how the treatment would compare with prior chemotherapy after the first consultation: 54.1% (vs 60.9%) thought it would be the same as chemotherapy, and 32.8% (vs 24.9%) thought that it would be better in comparison with the preclinic values (*P* = .015).

##### Commitment

 Perceptions about the number of hospital visits changed significantly, with 93% (vs 49.7% at the baseline) understanding that weekly visits were needed (*P* < .001). Moreover, the percentage of patients wanting to enter a phase 1 study increased to 84.3% from 71.5%, and this was significant (*P* < .001).

#### Patients' belief of a cancer cure

The final postconsultation question assessed patients' expectations about their cancer being cured in a phase 1 trial (Table 4). Three hundred patients replied to this question: 14.0% answered yes, 48.7% said no, and 37.3% did not know. For those who believed that they might be cured, the age distribution was similar; 33% and 67% were male and female, respectively (*P* = .17); 57% and 43% left formal education at an age < 17 years and at an age ≥ 17 years, respectively (*P* = .89); and 88% and 22% were referred by a cancer specialist and a nonspecialist, respectively. None of these factors were statistically significant.

## DISCUSSION

The characteristics of the study participants reflect the expected profile of patients attending the Drug Development Unit for age and cancer type,^4^, ^20^ with equal proportions leaving education before and after the age of 17 years. As expected for our unit, a cancer specialist or a general practitioner referred the majority of the patients.

The biggest motivators for our patients considering early‐stage clinical trials were the chance of tumor shrinkage and the paucity of alternative treatments; these findings were similar to those of a prior report.^8^ This highlights the fact that patients view phase 1 studies as an extension of previous conventional anticancer treatment.^17^ Phase 1 candidates tend to have better physical health and quality of life than those receiving best supportive care, and this may reflect on the higher expectation of a therapeutic benefit, as seen previously.^16^ Their referring physician's recommendation was important to half of participants, although older patients appeared to place more emphasis on this. The wishes of family were the least important factor in patients' decisions to participate. Older patients and those who had left formal education before the age of 17 years placed greater importance on their family's opinions. For the older group, this may reflect more reliance on family for support and assistance with traveling for frequent hospital appointments. Patients with gastrointestinal and “other” cancers were more likely to reply that they were interested in phase 1 trials because no other treatments were available; this was highly statistically significant. This could reflect that these tumor types may have fewer standard lines of treatment available in comparison with others such as breast and gynecological cancers.

Following from their motivations for considering trials, several themes related to perceptions of benefit, risks, and commitment were interesting. Patients had a high expectation of benefit from a phase 1 trial, with 43% believing that their tumor would shrink. This is more than double the reported response rates^1^, ^2^, ^3^ and represents a large discrepancy between expectations and what phase 1 trials offer. Although this is high for early clinical trials, these patients had more realistic expectations than those in a previous study in which 75% of patients expected a personal clinical benefit greater than 50%.^12^ The improved comprehension may reflect the current culture of better patient education in comparison with the study performed a decade ago. When considering risks, the majority of patients predicted moderate side effects comparable to those of chemotherapy. With respect to commitment, more than 70% of the patients expected to participate in a study, in agreement with previously published reports.^17^

When analyzing differences in perceptions after the first consultation, we found some statistically significant differences. Patient perceptions of benefits and risks remained largely unchanged. There was a more realistic perception of the time that patients would have to commit after the consultation. This, however, did not deter patients from considering a study, and paradoxically, a larger percentage of patients wished to enter a study after the consultation. Thus, our current first consultation in the phase 1 unit does not significantly change expectations but does clarify the understanding of the time commitment involved. These results will be reviewed by our focus groups to find ways to improve these discussions. It would be interesting to repeat this study at a later time point to assess how perceptions change after the receipt of treatment within a study.

Given the late stage in their cancer journey, more than half of the patients had not ruled out a cure; 14% believed that a cure was possible, and 37% were unsure. This likely represents human hope and is similar to the figures reported in 2 studies by Kass et al assessing phase 1 candidates.^15^, ^17^ It is tempting to interpret this as patient misinterpretation of previous discussions with healthcare professionals or a lack of information provided to patients throughout their cancer journey and to conclude that as a result we should inform our patients better.^11^ An alternative explanation is that this subset of patients, despite having been exposed to this information, have chosen to maintain unrealistic hope. The concept of therapeutic optimism has been reported before,^8^, ^14^, ^17^ and phase 1 physicians have to walk a fine line between reiterating a patient's poor prognosis, which may be seen as patronizing, and not disregarding the importance of human hope.^10^, ^11^, ^14^

Study limitations include the fact that only three‐quarters of the participants returned the postconsultation questionnaire. We aimed to minimize unreturned questionnaires by asking for the postconsultation responses to be completed in the clinic and thereby preventing loss or a failure to return them. Therefore, the missing postconsultation questionnaires (24%) likely represent patients who were unsuitable for early‐stage clinical trials because of an inappropriate clinical condition such as a poor performance status or deranged liver function. This cohort may have been disappointed by the clinical discussion, and the absence of their responses could have influenced the results. Alternatively, a lack of responses from patients who had decided against trial entry because of a lack of perceived personal benefit may have biased the returned‐questionnaire group. A second drawback is that no validated questionnaires were used to measure relevant patient characteristics such as quality of life or anxiety and depression scores. This would have been interesting to record, but when we were designing the study, we wanted a brief questionnaire to gain a snapshot of patients' perceptions and to maximize study participants' uptake. This is something that could be assessed in future studies. Lastly, because the consultations were not recorded, our assumption is that all information was shared with the patients attending the new phase 1 clinic in an understandable way, although recorded consultations in smaller studies have shown omissions during such discussions.^11^ However, without a content analysis of the consultations, we cannot ensure that this was the case. Workshops to train and appraise researchers consenting patients who are entering phase 1 studies have been shown to improve communications skills.^10^

In conclusion, we found that more than 80% of patients enrolling in early clinical oncology trials were motivated by the prospect of a clinical benefit. Approximately half the patients anticipated tumor shrinkage, and approximately a tenth still expected a cure. These rates conflict with the typical phase 1 response rates of 4% to 20%, and this discrepancy demonstrates the challenges facing patients and healthcare professionals during their interactions in phase 1 studies.

## FUNDING SUPPORT

This study was supported by a Cancer Research UK Cancer Centre grant to the Institute of Cancer Research and Experimental Cancer Medicine Centre and Biomedical Research Centre grants to the Institute of Cancer Research and The Royal Marsden.

## CONFLICT OF INTEREST DISCLOSURES

The authors made no disclosures.

## AUTHOR CONTRIBUTIONS

**Saoirse O. Dolly**: Conceptualization, methodology, software, validation, formal analysis, investigation, data curation, writing–original draft, writing–review and editing, visualization, supervision, project administration, and main contributor. **Eleftheria Kalaitzaki**: Conceptualization, methodology, software, validation, formal analysis, data curation, writing–original draft, writing–review and editing, visualization, supervision, and funding acquisition. **Martina Puglisi**: Conceptualization, methodology, software, formal analysis, investigation, data curation, writing–original draft, and writing–review and editing. **Sarah Stimpson**: Software, formal analysis, investigation, data curation, and writing–review and editing. **Janet Hanwell**: Conceptualization, formal analysis, investigation, data curation, and writing–review and editing. **Sonia Serrano Fandos**: Software, formal analysis, investigation, data curation, and writing–review and editing. **Sarah Stapleton**: Conceptualization, formal analysis, investigation, data curation, and writing–review and editing. **Thushara Ansari**: Software, investigation, data curation, and writing–review and editing. **Clare Peckitt**: Conceptualization, methodology, validation, formal analysis, data curation, writing–original draft, writing–review and editing, visualization, supervision, and funding acquisition. **Stan Kaye**: Conceptualization, validation, formal analysis, writing–review and editing, supervision, project administration, and funding acquisition. **Juanita Lopez**: Validation, formal analysis, writing–review and editing, supervision, project administration, and funding acquisition. **Timothy A. Yap**: Validation, formal analysis, writing–review and editing, supervision, project administration, and funding acquisition. **Winette van der Graaf**: Validation, formal analysis, writing–review and editing, supervision, project administration, and funding acquisition. **Johann de Bono**: Conceptualization, validation, formal analysis, writing–review and editing, supervision, project administration, and funding acquisition. **Udai Banerji**: Conceptualization, methodology, software, validation, formal analysis, data curation, writing–original draft, writing–review and editing, visualization, supervision, project administration, funding acquisition, and main contributor.

## Supporting information

Additional supporting information may be found in the online version of this article

Supporting Information Figure 1.

Supporting Information Figure 2.

Supporting Information Figure 3.
