# Supplementary figures and images for: A study of motivations and expectations of patients seen in phase 1 oncology clinics
Source: Cancer. 2016 Sep 26;122(22):3501–8. doi: 10.1002/cncr.30235 (PMC5111585; doi:10.1002/cncr.30235)

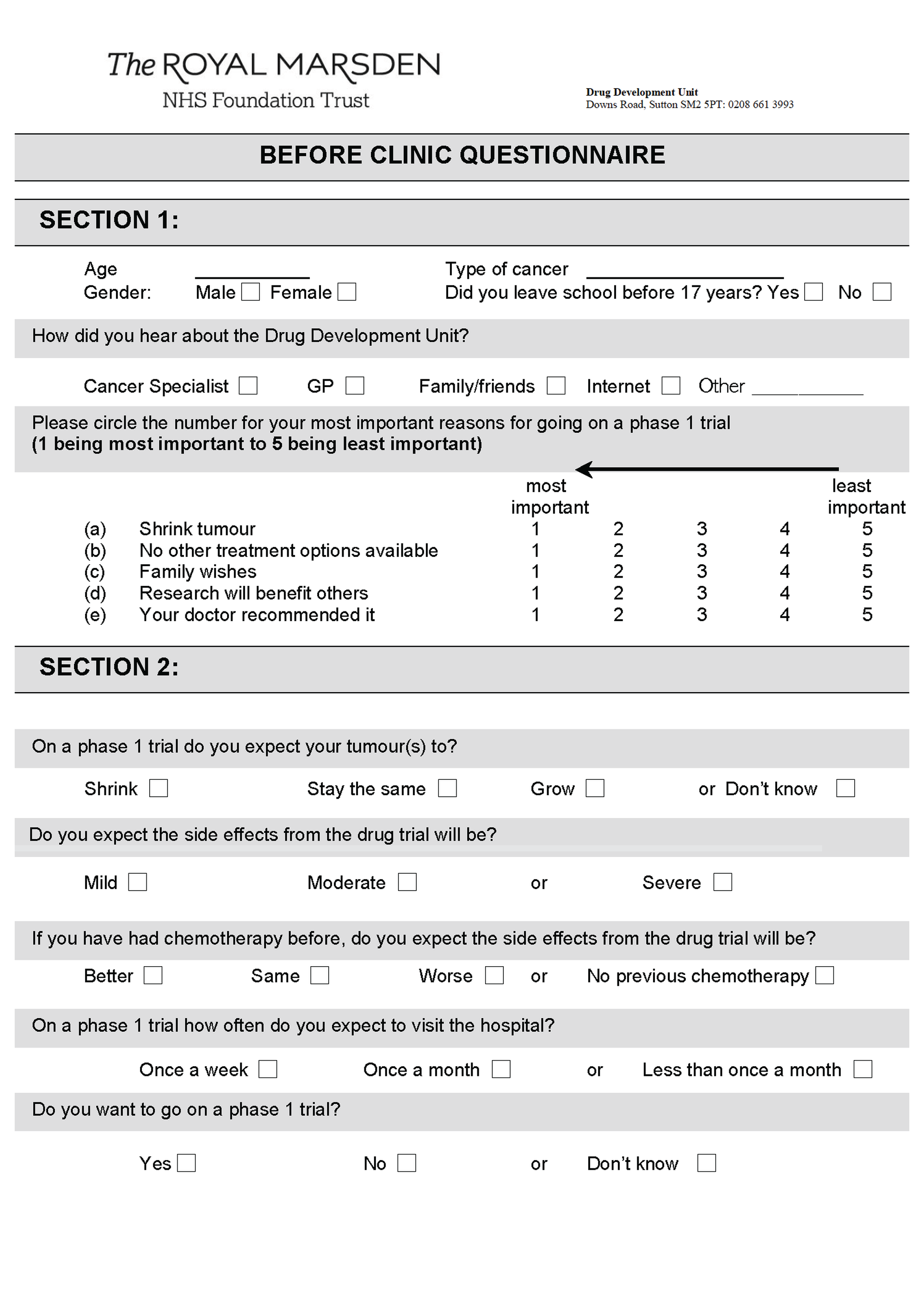

Supplement: Supplementary file 1 — Supporting Information Figure 1. [file CNCR-122-3501-s001.tif]

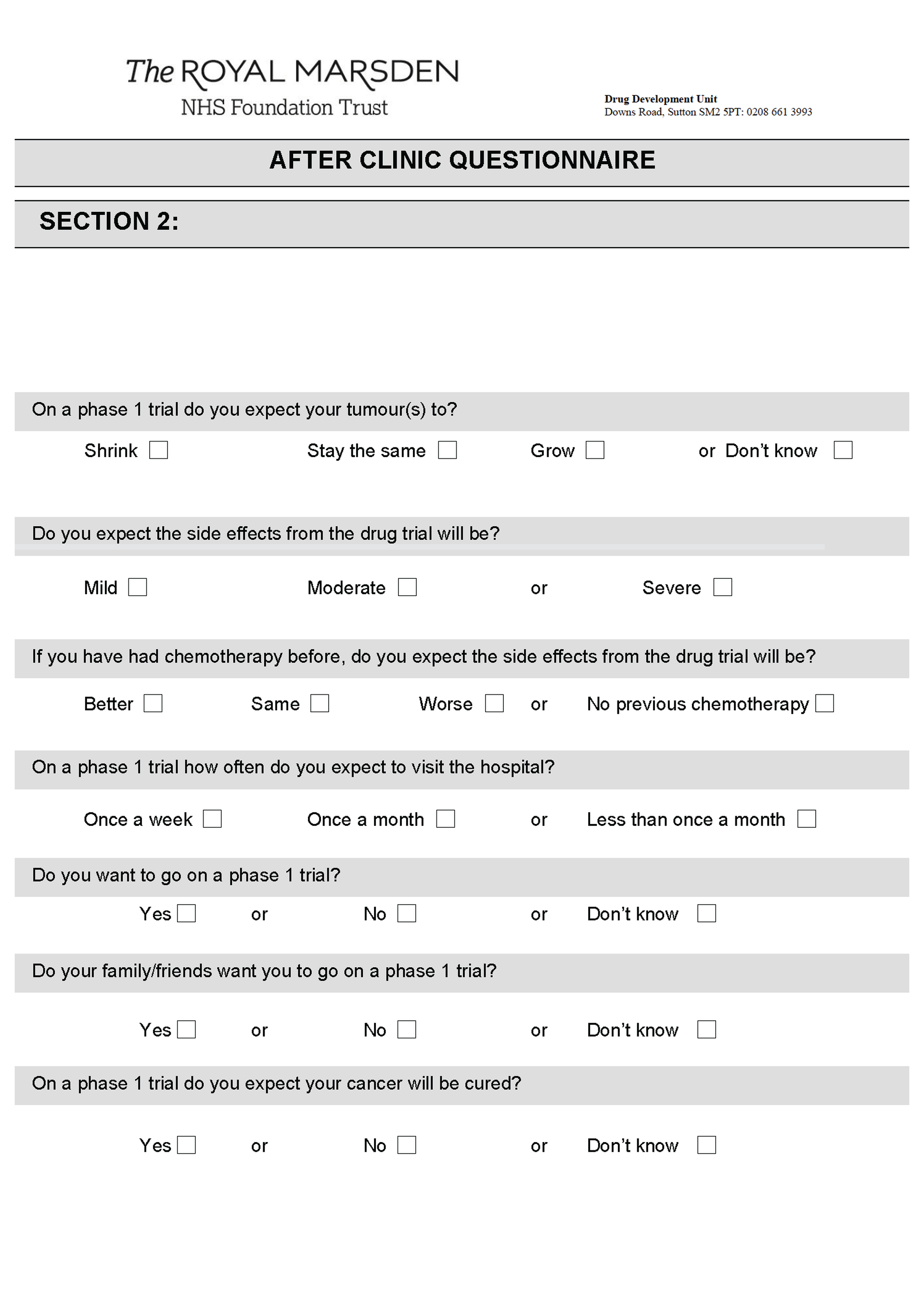

Supplement: Supplementary file 2 — Supporting Information Figure 2. [file CNCR-122-3501-s002.tif]

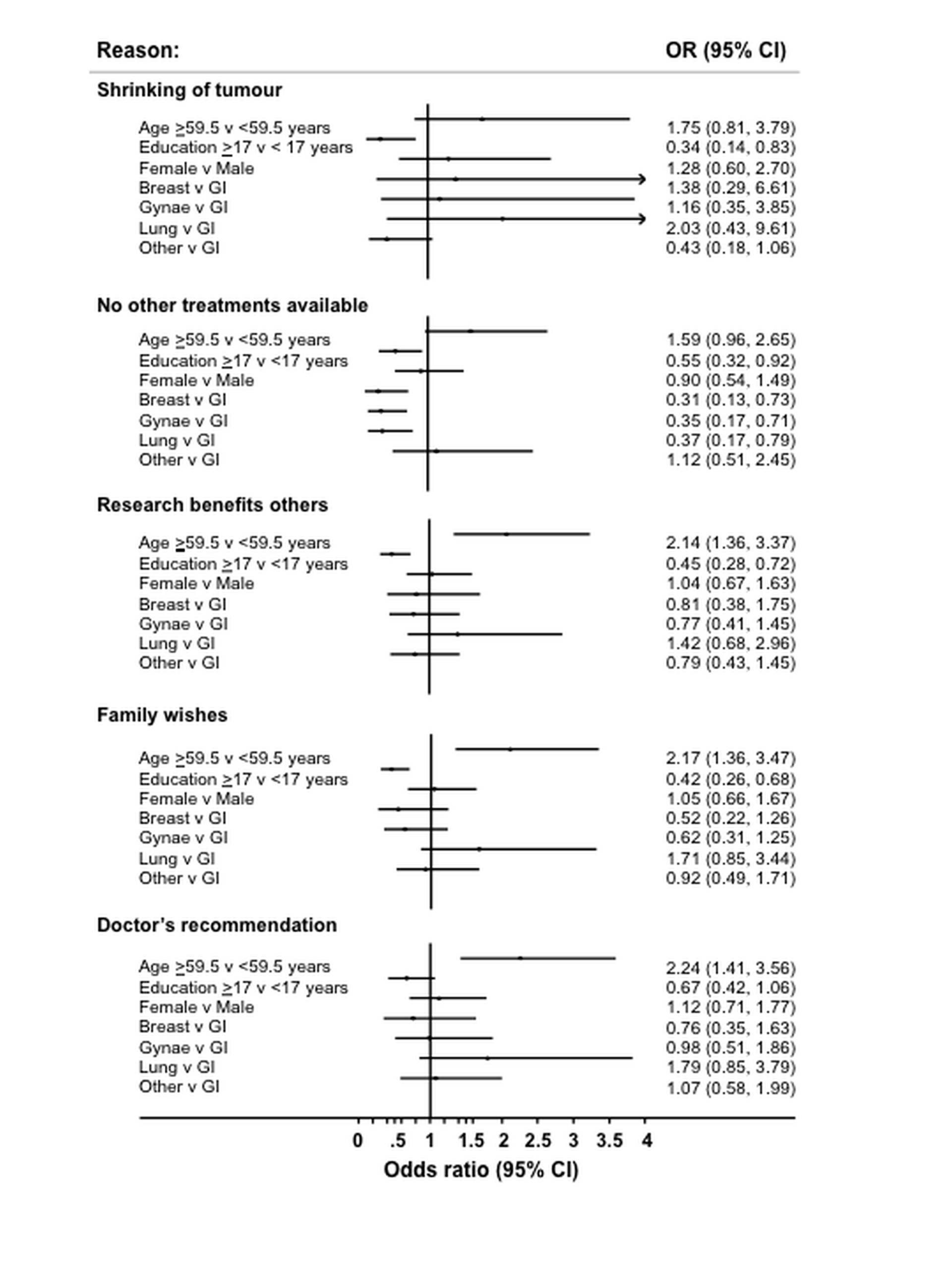

Supplement: Supplementary file 3 — Supporting Information Figure 3. [file CNCR-122-3501-s003.tif]
